# Mouth Movements as Possible Cues of Social Interest at Birth: New Evidences for Early Communicative Behaviors

**DOI:** 10.3389/fpsyg.2022.831733

**Published:** 2022-06-01

**Authors:** Bahia Guellai, Arlette Streri

**Affiliations:** ^1^LECD, Université Paris Nanterre, Institut Universitaire de France, Nanterre, France; ^2^INCC, Université Paris Cité, Paris, France

**Keywords:** neonates, motor feedback, interaction, face-to-face, imitation

## Abstract

Previous studies evidenced that different interactive contexts modulate the visual attention of newborns. In the present study, we investigated newborns' motor feedback as an additional cue to neonates' expression of interest. Using videos of interactive faces and a familiarization-test procedure, three different groups of newborns were assigned to three different conditions (i.e., one condition with a talking face during familiarization and silently moving faces at test, silently moving/silently moving condition, or talking/static condition). Following studies on neonatal imitation, mouth movements were analyzed as indicators of social interest. We expected the occurrence of mouth movements in the newborns to differ according to different conditions: (a) whether or not the face in front of them was talking and (b) if the person had been already seen or was new. Results revealed that a talking face elicited more motor feedback from the newborns than a silent one and that there was no difference in front of the familiar face or the novel one. Finally, frequencies of mouth movements were greater, and latencies of appearance of the first mouth movement were shorter, in front of a static vs. a dynamic face. These results are congruent with the idea of the existence of “a sense” for interaction at birth, and therefore new approaches in newborn studies are discussed.

## Introduction

For centuries, one of the central questions of philosophers and scientists has been focused on understanding what makes humans so special? Many authors would now claim that this is due to our capacity to interact with each other using a complex system of communication using both verbal and non-verbal cues, which makes us unique. Indeed, in everyday life, humans almost constantly interact which each other, and most of these interactions occur in face-to-face contexts. In these contexts, not only the face but also the whole body becomes powerful vectors of communication (Bruce and Young, 2000). While abundant literature exists on how adults interact with each other, less is known about the development of this capacity in the early stages (Gratier and Trevarthen, 2008; Gratier et al., 2015; Dominguez et al., 2016). In the present study, we investigated the emergence of socio-communicative behaviors during the neonatal period.

From birth, and even before birth, human infants are surrounded by socio-communicative cues. Two of these cues are speech and faces. While already in the womb, the fetus hears voices in its surrounding environment (DeCasper et al., 1994), it is only from birth that the newborn can see and hear faces talking. In the past decades or so, the abilities of newborns to process speech and faces have been studied separately. It is now known that newborns' attention is tuned to speech (Vouloumanos and Werker, 2007; Vouloumanos et al., 2010) and that newborns already have some auditory preferences, such as listening to their mother's voice compared to a stranger's one (DeCasper and Fifer, 1980) or to their native language when compared to non-native ones (Mehler et al., 1988; Moon et al., 1993). Moreover, despite a weak visual system (Braddick and Atkinson, 2011), newborns can learn and recognize their live mother's face (Field et al., 1984; Bushnell et al., 1989; Pascalis et al., 1995) and unfamiliar faces presented under photographs (Pascalis and de Schonen, 1994; Turati et al., 2006, 2008; Gava et al., 2008).

While these studies shed light on remarkable feats of the newborn infant, they did not consider talking faces as a unit and therefore the possible interactions between speech and face processing at birth. To our knowledge, only a few studies investigated this possibility. In a study (Sai, 2005), authors encouraged a group of mothers to talk to their infants immediately after birth till the test session (i.e., occurring on average 7 h later), while another group was asked not to interact with them verbally. In the test session, when the mother's face and a stranger's face were presented side-by-side, the newborns looked longer and oriented more to their mother's face than to a stranger's face only if their mother had previously talked to them. The author concluded that experience with both the mother's voice and her face during the first hours after birth enhanced newborn's encoding of their mother's face. However, because fetuses hear their mother's voice and prefer it at birth (DeCasper and Spence, 1986), it is possible that in Sai's experiments (Sai, 2005) newborns who received verbal interaction, associated with socio-communicative cues such as direct eye gaze, were reinforced soon after birth, and that this reinforcement helped them to encode and memorize their mother's face.

**Figure 1:**
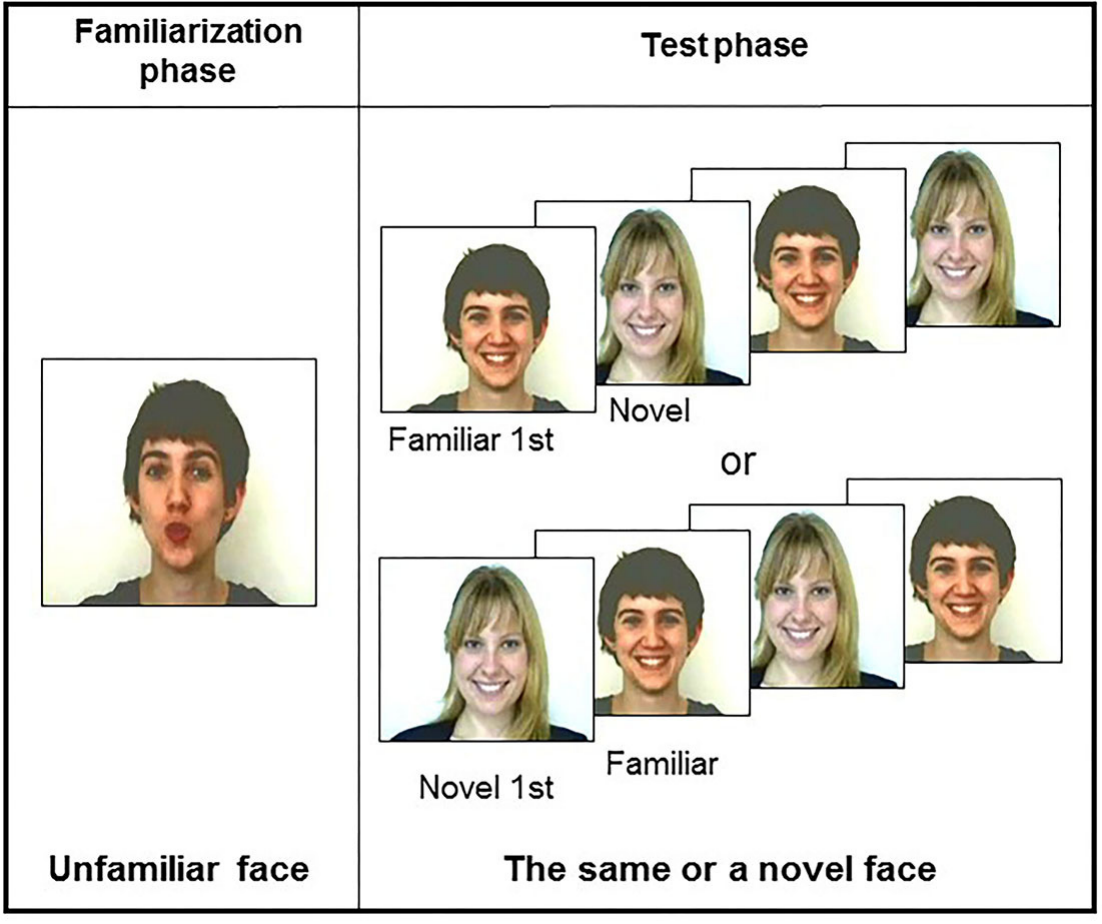
Presentation of the procedure used for the three conditions.

This possibility has been called into question in more recent studies (Coulon et al., 2011; Guellaï and Streri, 2011; Guellaï et al., 2011, 2020). Using a familiarization-test procedure, and, for the first time, videos of dynamic unfamiliar faces, the authors proposed different conditions for newborn infants. Each condition was presented to one group of newborns. In a first study (Coulon et al., 2011), infants saw during the familiarization phase either the video of a woman's face talking to them or with her lips moving but no speech sounds. Then, at the test session, they saw the photographs of the same face (i.e., familiar) or a new one. Analyses of looking times at test showed that the majority of newborns elicited a visual preference for the familiar over the new face only when the face was seen talking during the familiarization phase. To further explore the interactions between speech and facial cues, additional conditions were tested in other studies (Guellaï and Streri, 2011; Guellaï et al., 2011, 2020) (Figure 1). Interestingly, results evidenced that newborns recognized and preferred to look at a previously seen face only when this person talked to them with direct gaze during the familiarization phase, and when the person was seen under photographs or talking again at the test. In other words, the interactive face-to-face situation is of particular interest to studying newborns' encoding abilities of unfamiliar persons. More recently, it has been evidenced that no matter which language (i.e., native or non-native) is used by the talking face during the familiarization phase, it is the audiovisual congruent situation that is important for newborns to encode and later show a visual preference for someone who talked to them (Guellai et al., 2015).

These results evidenced that newborns already elicit a strong visual preference for persons who interacted with them verbally and that this goes beyond the mother's face. Nonetheless, one of the limits of this set of studies is that it focused on newborns' visual attention as the main cue of social interest, whereas other cues such as newborns' motor feedback could constitute additional indicators of newborns' expression of interest. In that sense, the use of videos of faces is interesting to control for different factors, such as the characteristics of the acoustic signal or the timing of the presentations. One possibility could be that the mouth movements of newborns are informative of social interest and therefore may vary depending on the interactive situations presented (i.e., talking faces, silent dynamic faces, or static faces). Indeed, some authors evidenced that the behaviors of older infants vary according to the face-to-face situation proposed (Tronick et al., 1979). Using a specific paradigm called “the still-face paradigm,” these authors proposed a live face-to-face interaction between an infant and an adult, interspersed with a period in which the adult suddenly becomes unresponsive and poses a stationary neutral face while maintaining eye contact. Infants at 2 months of age react to the adults' unresponsiveness during the still-face period with decreased visual attention and positive affect (Lamb et al., 1987). Such results are interpreted in terms of infants' affective attunement to social patterns and rudimentary expectations about the nature of face-to-face interactions (Muir and Hains, 1993). Nonetheless, to date, no study was interested in looking at newborns' facial motor feedback as serving communicative functions when presented with different face-to-face interactive situations. To identify newborns' facial gestures, we will consider those mouth movements that have been widely explored in the light of neonatal imitation (Meltzoff and Moore, 1977, 1994; Nagy et al., 2005, 2020).

Indeed, there already seem to be connections between newborns' orofacial motor capabilities and auditory and visual face information. Concerning newborns' feedback, it is well-established that newborn infants can imitate faces in the first few hours after birth, demonstrating a link between orofacial motor control and visual face perception. Other studies evidenced that newborns are able to match auditory information to motor actions. For example, they produce more mouth openings when listening to /a/ vs. /m/ sounds, and they produce more mouth closing when listening to /m/ vs. /a/ sounds (Chen et al., 2004). Moreover, facial imitation is more robust at birth in the presence of congruent (as opposed to incongruent) audiovisual speech: infants will produce more mouth openings when presented with a face saying /a/ than with the face alone, or that face dubbed with an /i/ audio track (Coulon et al., 2013).

The present study aimed at addressing two questions: (a) is there any difference in newborns' attention and orofacial motor feedback when they are facing someone talking to them or looking at them silently? (b) Would they elicit more mouth movements in front of a familiar vs. an unfamiliar person? Newborns were tested in three different conditions. Following previous studies, the first group of newborns (i.e., the talking/silently moving condition) was first familiarized with a woman talking to them in an infant-directed speech style. Then, in a test phase, they saw the familiar and a new person looking at them silently moving. The second group of newborns (i.e., the silently moving/silently moving condition) was familiarized with a woman silently moving while looking at them; at the test, they saw the same woman and a new one still silently moving while looking at them. Finally, as studies on neonatal imitation or face processing at birth used static presentations of faces during the test session, the third group of newborns was familiarized with a talking face and then presented with the photographs of the familiar and new faces at the test (i.e., the talking/static condition). We wanted to see if the face-to-face interactive situations proposed to the newborns would modulate their behaviors, in particular their mouth movements, during and after the familiarization period. Following the results of studies on neonatal imitation both in humans (Reissland, 1988; Coulon et al., 2013; Nagy et al., 2020) and non-human primates (Simpson et al., 2013), we analyzed newborns' facial behaviors as potential indicators of social interaction at birth. We expected newborns (a) to elicit more mouth movements in front of a talking vs. a silent person and (b) in front of a familiar vs. an unfamiliar person.

## Methods

### Participants

The participants were 36 full-term newborns (18 girls) from the maternity hospital of Bichat in Paris. All newborns were in good health (APGAR scores above 8). The mean age was 56.4 h (range: 18–98 h). Newborns whose mothers had major complications during pregnancy and those with medical problems were systematically excluded from the study. An additional 22 newborns (10 girls; age range: 22–100 h) were excluded from the original sample because of fussiness (*n* = 16), or sleepiness (*n* = 4), or experimental errors (*n* = 2). The rejection criteria were assessed by two different experimenters.

### Apparatus

Newborns were observed in a quiet room where they had been previously brought by one or both parents. Before testing, we systematically ensured that parents and medical staff gave their consent to participate in the study. Each newborn was positioned in a semi-upright position (30°) in an adapted rigid seat. The seat was placed on a table facing a 19-inch DELL color monitor, 35 cm away from the infant's eyes. Two speakers were placed on each side of the DELL monitor. A first experimenter (Experimenter 1) always stood behind the newborn during the whole session to monitor for potential signs of discomfort. A small video camera was directed at the newborn and recorded the whole experiment (the temporal resolution was 25 images/s). Images were retransmitted on two video monitors. One allowed a second experimenter (Experimenter 2) to code the duration of looking. The other allowed the parents to see their baby. The parents sat behind and far from the baby, so that the infant could not see them. Parents were instructed to not intervene (speak or come near their baby) during the whole experiment.

### Stimuli

Color video films of two female faces were recorded. These videos were recorded under the same lighting conditions (mean: 16 cd/m^2^) with the same white background in a soundproof room. The video framing took into account the faces of females from their top heads to their shoulders. The two women differed in terms of eye and hair color and style: short brown hair and brown eyes (brown-haired face) vs. long blond hair and green eyes (blonde face) (Figure 1). We chose two different faces of females so that by counterbalancing their presentation across subjects, we ensured that the results found were not due to the physical characteristics of the stimuli. Two different recordings were realized: in the first recording, each woman looked directly at the camera and addressed the newborn in the following way: “*Hello baby, how are you? Are you okay? Yes, I know, just a few hours after your birth, and we're already asking you to do things. You know, for us, it is very important to study the early behaviors of newborns like you....”* We ensured that sound intensities at the speakers in the testing room were identical for both stimuli (mean: 65 dB). In a second recording, each woman looked at the camera without talking but silently moving (i.e., translation movements of the whole head). Each of the videos created lasted for 90 s and was used in the familiarization phase.

For the test phase, either silent videos or photographs were presented (Figure 1). We applied the same lighting conditions to all the images (i.e., mean: 16 cd/m^2^). The maximal length of each image presentation for the test phase was 60 s. Each facial image subtended about 30° of visual angle horizontally and vertically on the color monitor.

### Design and Procedure

The experiment began as the infant was seated. The familiarization-test procedure was similar to the experiments conducted by Guellaï et al. The familiarization phase started with the presentation of one of the two female faces talking continuously for 90 s. Immediately after the familiarization phase, the test phase began, where the newborns saw the familiar face and a new one twice successively and in alternation. The order of presentation of the two faces in the test phase, familiar first or new first, was randomly counterbalanced across subjects by a computer program.

During the familiarization phase, Experimenter 2, unaware of the face presented, pressed and held a key button on a computer keyboard when the infant looked at the screen and released it when the infant looked away. The computer program recorded the accumulated looking times. During the test phase, Experimenter 2 proceeded in the same way, but when the newborns looked away from the screen for more than 2 s, the computer program automatically switched to the next face. A switch also occurred after newborns had looked at the face continuously for 60 s (i.e., the maximum length of each video in the test phase). The computer program also required a minimum looking time of 2 s at the screen.

**Figure 2:**
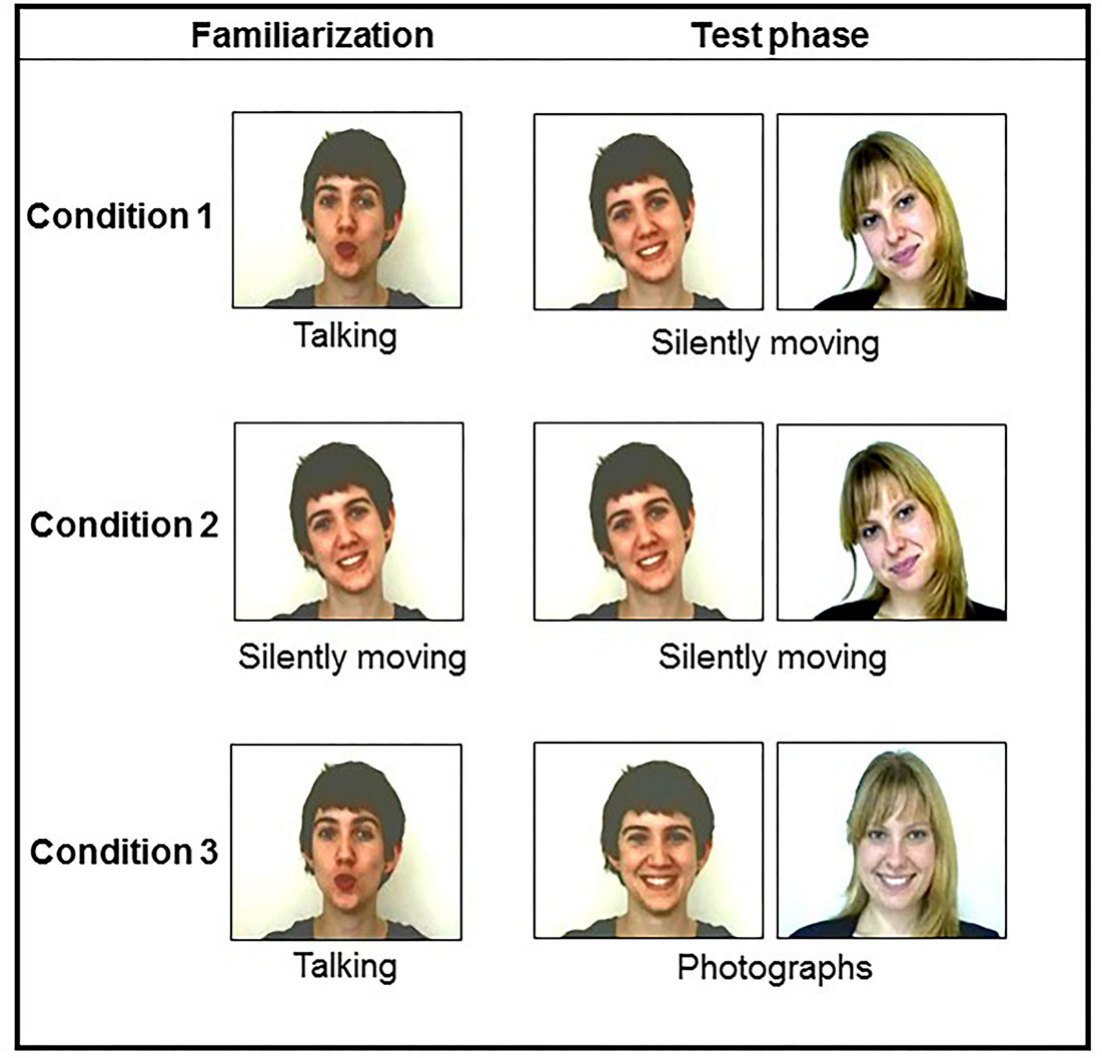
Examples of the stimuli presented for each condition.

Twelve newborns looked at the talking/silently moving condition, 12 others looked at the silently moving/silently moving one, and 12 additional ones looked at the talking/photograph condition. For each familiarization condition, half of the newborns saw the blonde woman and the other half the brown-haired one (Figure 2).

### Data Analysis

The facial gestures of infants in the familiarization and test phases were analyzed off-line, frame-by-frame (30 frames per second) from the videos, using the Noldus Observer XT (Noldus, Wageningen, the Netherlands). Two coders, blind to the condition, scored all the occurrences of facial gestures produced by infants: mouth opening (MO), tongue protrusion (TP), lip protrusion (LP), and lip spreading (LS). Thus, a frequency corresponding to the number of mouth movements per second was defined. The mouth movements were chosen and defined according to the previous studies that investigated the neonate's behavioral feedback in imitative situations (Reissland, 1988; Coulon et al., 2013; Simpson et al., 2013). MO was operationally defined as a high-frequency opening and closing of the mouth in which the lips parted and rejoined within 2 s. TP was operationally defined as a clear forward thrust of the tongue in which the tongue protruded beyond the lips. LP was operationally defined as a clear forward thrust of the lips. LS was operationally defined as the lateral broadening of the lips and returning to their resting position within 2 s. Other behaviors, such as cough, hiccups, etc., which could involve motor actions, such as mouth opening or lip spreading, were coded but not considered in the analysis. Moreover, for each mouth movement, latencies were also considered. We expected differences in the frequencies and latencies of the overall mouth movements depending on the different conditions proposed and not necessarily on specific mouth movements. Statistical analyses were therefore performed on overall mouth movements and not on specific movements. Indeed, as presented earlier, we expected newborns to elicit more mouth movements in front of a talking vs. a silent person and in front of a familiar vs. an unfamiliar person. Finally, we also took into account infants' looking times to the stimuli, but these data are already presented elsewhere (Coulon et al., 2011; Guellaï et al., 2011). Herein, we will only present the looking times for the familiarization phase as an index of infants' visual attention. The coding comparison was made action by action (MO, TP, LP, LS, and looking times) to ensure that the two different coders scored the same event at the same time during the familiarization and the four presentations of the test phases. Observers were blind to the stimulus, and inter-observers' reliability was high throughout all the experiments (Pearson's *r* ≥ 0.9). Statistical analyses were conducted using the STATISTICA 14.0 software.

## Results

The looking times toward the faces and overall mouth movements were taken as the dependent measure. We first checked for the normal distribution of the looking times in each condition (Kolmogorov–Smirnov test *p* > 0.5), and later also for the frequencies of mouth movements (KS test, *p* > 0.3) and for the latencies of the first mouth movements (KS test, *p* > 0.3) for each condition during the familiarization phase. Results showed that distributions did not differ significantly from normal through KS tests. We also checked for the normal distribution of the data during the test phase for the looking times for each condition (KS test, *p* > 0.6), for the frequencies of mouth movements in each condition (KS test, *p* > 0.2), and for the latencies of the first mouth movement (KS test, *p* > 0.4). Face presentation looking times, frequencies, and latencies of mouth movements recorded during the test phase followed a normal distribution.

### Familiarization Phase

#### Visual Attention

In the familiarization phase, the newborns looked at the video for an average of 73.7 s (*SE* = 5.7) for the talking/silently moving condition; for 72 s (*SE* = 2.7) in the silently moving/silently moving condition; and for 68 s (*SE* = 4.4) in the talking/static one. A three-way (condition: talking/silently moving, silently moving/silent, or talking/static) ANOVA was performed on these looking times. The results revealed no effect of the condition on the looking times [*F*_(2,33)_ = 1.54, *p* = 0.2, η^2^ = 0.9]. Overall, newborns looked at the videos for an equal amount of time in each condition during the familiarization phase. No other effect or interaction was significant.

#### Facial Gesture Frequencies

**Figure 3:**
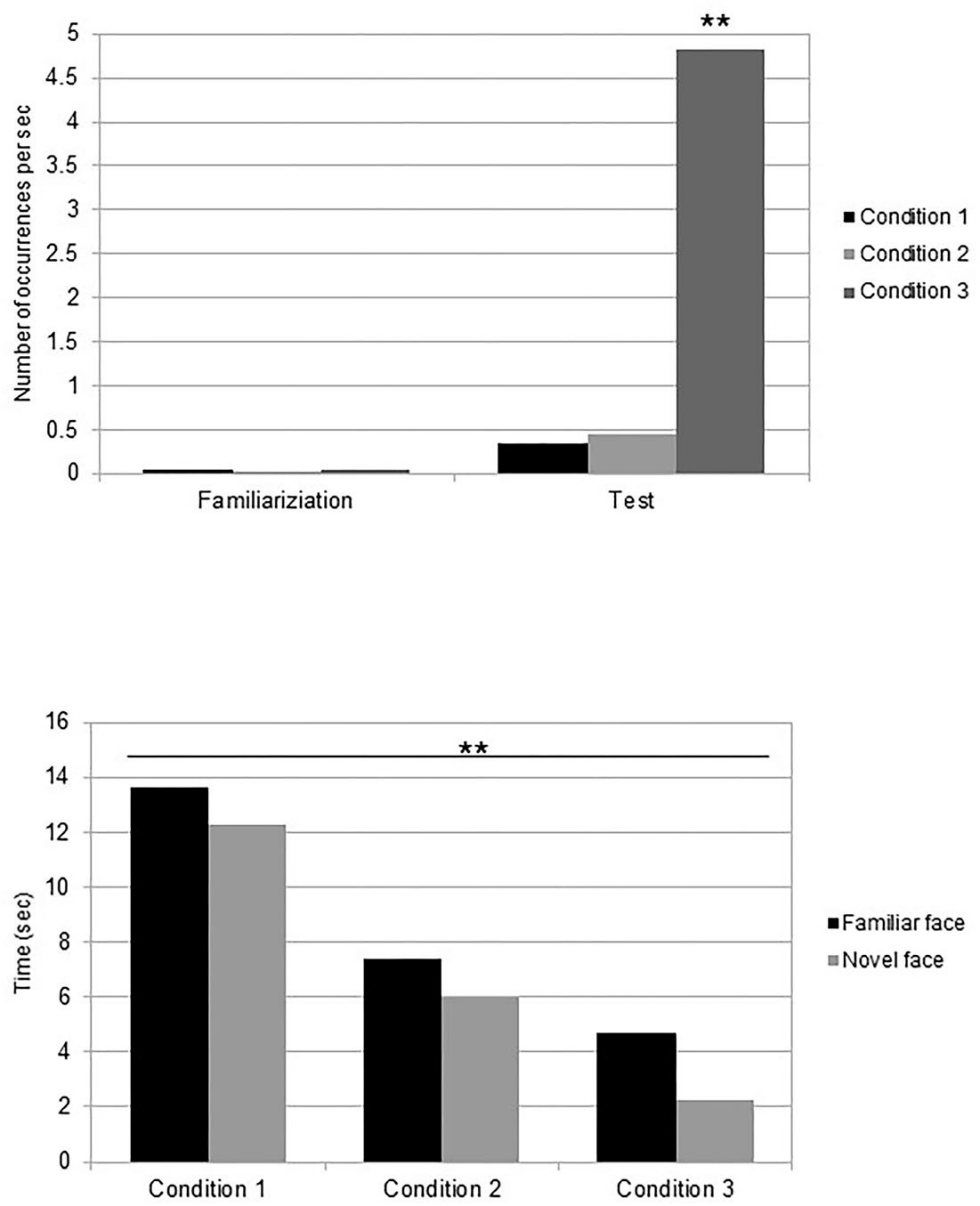
Mean frequencies of mouth movements during the familiarization and test phases for each condition, and latencies of appearance of the first mouth movement in the test phase in front of the familiar and novel faces. **p < 0.05.

The number of occurrences of facial gestures per second during the familiarization phase was analyzed. The average frequency of newborns' mouth movements was *M* = 0.05 (*SE* = 0.002) for the talking/silently condition, *M* = 0.03 (*SE* = 0.002) for the silently moving/silently moving condition, and *M* = 0.05 (*SE* = 0.002) for the talking/static condition. A three-way (condition: talking/silently moving, silently moving/silent, or talking/static) ANOVA was performed on the frequency of overall mouth movements. The results revealed no effect of condition [*F*_(2,33)_ = 2.37, *p* = 0.11, η^2^ = 0.13]. Nevertheless, *post-hoc* analyses revealed than when comparing the familiarization conditions of both the talking faces to the silently moving face condition, newborns who looked at a talking face during the familiarization performed more mouth movements (M = 0.051, *SE* = 0.003) than those who looked at a silently moving face (M = 0.031, *SE* = 0.002) [*t*_(22)_ = 1.83, *p* = 0.03, Cohen's d = 0.7] (Figure 3).

Taken together, analysis of the looking times and the mouth movements during the familiarization phase evidenced a consistency in the visual attention of newborns when presented either with videos of talking faces or with a person looking at them silently. Nonetheless, it appears that they performed more mouth gestures in front of a talking vs. a silent face.

### Test Phase

#### Visual Attention

The looking times (seconds) of newborns at the test in front of the familiar and the new face for each condition are presented in Table 1. As previously reported, newborns looked more at the familiar face only in the talking/static condition (Guellaï et al., 2011).

**Table 1:** Mean looking times in seconds in front of the familiar and new faces at test phase.

**Condition 1**		**Condition 2**		**Condition 3**	
*Talking/*		*Silently moving/*		*Talking/*	
*Silently moving*		*Silently moving*		*Static*	
**Familiar**	**New**	**Familiar**	**New**	**Familiar**	**New**
30.3	32	29.7	33.8	^**^ 40.6	22.7
(0.54)	(0.58)	(7.6)	(9)	(0.47)	(0.45)

*Standard errors are indicated in brackets*.

** *p < 0.01*.

#### Frequencies of Mouth Movements

A 3 (condition: talking/silently moving, silently moving/silently moving, or talking/static) × 2 (familiarization face: blonde or brown-haired) × 2 (block of presentation: F1N1 or N1F1, F2N2 or N2F2) × 2 (test: blonde or brown-haired) ANOVA was performed on the overall frequencies with the two last factors within subjects. The analysis revealed a significant effect of the condition [*F*_(4,58)_ = 3.69, *p* = 0.009, η^2^ = 0.20]. No other effect or interaction was significant.

*Post-hoc* analysis revealed that newborns in the talking/static condition performed more mouth movements (*M* = 4.83, *SE* = 0.54) than those in the talking/silently moving condition [*M* = 0.33, *SE* = 0.04; *t*_(22)_ = −2.4, *p* = 0.03, Cohen's d = −0.8] and those in the silently moving/silently moving condition [*M* = 0.46, *SE* = 0.06; *t*_(22)_ = 2.33, *p* = 0.03, Cohen's d = 1]. No difference was observed between the two conditions with the silently moving face at test session [*t*_(22)_ = −0.5, *p* = 0.62, Cohen's d = −0.2] (Figure 3). Further analysis showed that the frequency of mouth movements was not different in front of the familiar face (*M* = 0.91, *SE* = 0.07) or in front of the novel one [*M* = 0.97, *SE* = 0.05, *t*_(35)_ = −0.17, *p* = 0.87, Cohen's d = −0.05] across conditions.

Finally, when comparing frequencies of mouth movements in the familiarization and test phases, newborns performed more mouth movements in the test phase than in the familiarization phase in the talking/silently moving [two-tailed *t*-test *t*_(11)_ = 2.36, *p* = 0.038, Cohen's d = 1.4] and in the talking/static [*t*_(11)_ = 2.56, *p* = 0.026, Cohen's d = 1.5] conditions, but not in the silently moving/silently moving condition [*t*_(11)_ = 2.11, *p* = 0.06, Cohen's d = 1.2] (Figure 3).

#### Reaction Times

The reaction time was defined as the delay between the appearance of the familiar or novel faces on the screen at the test and the appearance of the first mouth movement of the newborns. In front of the familiar face, newborns in the talking/silently moving condition realized their first mouth movement on average after 13.63 s (*SE* = 1.5), after 7.38 s (*SE* = 0.6) in the silently moving/silently moving condition, and in the talking/static condition it occurred after 4.67 s (*SE* = 0.7). In front of the novel face, newborns in the talking/silently moving condition realized their first mouth movement on average after 12.25 s (*SE* = 1.3), after 6.00 s (*SE* = 0.5) in the silently moving/silently moving condition, and in the talking/static condition it occurred after 2.25 s (*SE* = 0.4). We performed a 3 (condition: talking/silently moving, silently moving/silently moving, or talking/static) × 2 (familiar vs. novel face) ANOVA on reaction times at the test. The results revealed a significant effect of condition [*F*_(4,64)_ = 3.80, *p* = 0.008, η_2_ = 0.19]. No other effect or interaction was significant (Figure 3). *Post-hoc* analysis evidenced that overall mouth movements appeared significantly quicker in the talking/static condition than in the other conditions [*t*-test, *t*_(11)_ = 1.46, *p* = 0.04, Cohen's d = 0.9].

## Discussion

The present study aimed at exploring the behavioral feedback of newborns in different interactive situations using videos of faces. Following studies on neonatal imitation, mouth movements were analyzed as possible indicators of communicative behaviors. More precisely, we expected newborns' occurrences of mouth movements to differ according to the different conditions: (a) whether or not the person in front of them was talking and (b) if the person had been already seen or was a new one.

Overall, our results show that the context of presentation of a potential social partner affects the gesture rates and latencies of infants, suggesting that newborns are already sensitive to the conditions of presentation of unfamiliar faces. More precisely, newborns produced more mouth movements in front of someone who was talking to them rather than someone looking at them silently moving. Besides, they produced more mouth movements following familiarization with a talking vs. a silent face. In other words, this first result shows that a talking face elicits more motor feedback from the newborns than a silent face. This extends results of previous studies showing that the production of specific mouth movements by newborns occurred more in front of congruent audiovisual presentations of faces than incongruent ones (Chen et al., 2004; Coulon et al., 2013). Nonetheless, whereas in the past studies, faces were repeating the same speech sound (i.e., a vowel or a consonant), in the present study, we presented continuously talking faces closer to real-life situations. It is therefore possible that verbal interactive situations are favorable to eliciting what could be seen as precursors of communicative actions in the neonatal period (Trevarthen, 1998; Dominguez et al., 2016).

Moreover, our results did not evidence a difference in the behaviors of newborns in front of the familiar face or the novel one. Therefore, soon after birth, infants do not appear to use imitation for the purpose of identifying social partners. This result is surprising as previous work on imitation has associated neonates' production (and reproduction) of mouth movements as indicators of social partner recognition (Meltzoff and Moore, 1994; Meltzoff et al., 2018; Nagy et al., 2020). Nonetheless, previous studies did not use the familiarity–novelty procedure as they presented either the familiar face (i.e., the mother) or the stranger's one during a unique interactive test period. To our knowledge, only one study used the familiarity–novelty procedure to explore the imitation of non-human primate neonates (Simpson et al., 2013). Results evidenced that rhesus macaques in the first week of life do not appear to produce more mouth movements in front of a familiar or a novel face. Here, in the talking/ static condition, newborns' visual attention and their motor feedback do not have the same pattern of results. Whereas visual attention indicates a preference for the familiar face, motor feedback does not. This suggests that considering different types of dependent variables may be complementary and useful for a better understanding of the infants' behavior, particularly in the studies on newborns. Further studies with a more systematic analysis of the complementary dependent variables should be conducted in the future.

Finally, when comparing newborns' production of mouth movements during the test phase, different results were revealed. First, it appeared that frequencies of mouth movements were greater in the test phase than during the familiarization phase. This could be explained by the fact that a delay is usually observed in the neonatal motor feedback with mouth movements appearing after the face began producing it (Coulon et al., 2013). Second, frequencies of mouth movements were greater, and latencies of appearance of the first movement were shorter, in front of a static vs. a dynamic face. This latter result could be interpreted as evidence of newborns' ability to discriminate between photographs and videos of faces similar to that observed in older infants (Hunnius and Geuze, 2004). Our results have also some parallels with previous studies using the still-face paradigm (Tronick et al., 1979). In this paradigm, a normal face-to-face interaction between an infant and an adult is interspersed with a period in which the adult suddenly becomes unresponsive and poses a stationary neutral face while maintaining eye contact. Infants as young as 2 months of age react to the adults' unresponsiveness during the still-face period with decreased visual attention and positive affect. Such results are interpreted in terms of infants' affective attunement to social patterns and rudimentary expectations about the nature of face-to-face interactions. Some studies using this paradigm with younger infants did not find any difference in the infants' reaction in front of an interactive or a static face (Bertin and Striano, 2006). This latter result is different from that observed in the present study. A possibility is that in the previously cited study, the authors analyzed the gaze and smiles of newborns, whereas here we analyzed a broader range of mouth movements replicating those investigated in the imitation studies. A possible explanation for the greater frequencies of newborns' mouth movements observed in front of a static face compared to a dynamic one could be that infants are trying to make the face react. Another possibility is that attention to the face is enhanced when the person is talking, which would attract newborns' attention and therefore would lead to less orofacial movements. Indeed, some studies evidenced that multimodal presentation enhances attention in very young infants (Spelke, 1976).

To conclude, the results of the present study are congruent with the idea of the existence of precursors of effective and positive interactions as soon as birth. Further work is needed to explore the development of infants' feedback in different interactive situations, as this could have important implications for neonatal care units.

## Data Availability Statement

The raw data supporting the conclusions of this article will be made available by the authors, without undue reservation.

## Ethics Statement

The studies involving human participants were reviewed and approved by CER Paris Nanterre. Written informed consent to participate in this study was provided by the participants' legal guardian/next of kin. Written informed consent was obtained from the individual(s) for the publication of any potentially identifiable images or data included in this article.

## Author Contributions

BG collected and analyzed data. BG and AS wrote article. Both authors contributed to the article and approved the submitted version.

## Conflict of Interest

The authors declare that the research was conducted in the absence of any commercial or financial relationships that could be construed as a potential conflict of interest.

## Publisher's Note

All claims expressed in this article are solely those of the authors and do not necessarily represent those of their affiliated organizations, or those of the publisher, the editors and the reviewers. Any product that may be evaluated in this article, or claim that may be made by its manufacturer, is not guaranteed or endorsed by the publisher.
